# Caring for the whole person: transgender‐competent HIV pre‐exposure prophylaxis as part of integrated primary healthcare services in Vietnam

**DOI:** 10.1002/jia2.25996

**Published:** 2022-10-12

**Authors:** Anh H. Doan, Chau M. H. Vu, Thu T. Nguyen, Kimberly E. Green, Huong T. T. Phan, Rena Janamnuaysook, Bao N. Vu, Thanh M. Le, Khang Q. Do, Tham T. Tran, Trang M. Ngo, Lopa Basu, Long K. Tran, Zoe Humeau

**Affiliations:** ^1^ PATH Hanoi Vietnam; ^2^ Vietnam Network of Transgender People Hanoi Vietnam; ^3^ My Home Ho Chi Minh City Vietnam; ^4^ Vietnam Administration for HIV/AIDS Control Ministry of Health Hanoi Vietnam; ^5^ Institute of HIV Research and Innovation Bangkok Thailand; ^6^ Glink Ho Chi Minh City Vietnam; ^7^ Galant Ho Chi Minh City Vietnam; ^8^ US Agency for International Development Hanoi Vietnam

**Keywords:** transgender women, gender‐affirming care, HIV, PrEP, primary healthcare, Vietnam

## Abstract

**Introduction:**

Although HIV prevalence among transgender women who have sex with men in Vietnam is high (16–18%), uptake of pre‐exposure prophylaxis (PrEP) is low compared to other populations. When PrEP was initiated in 2017, gender‐affirming healthcare was largely unavailable. Lack of access to competent, stigma‐free healthcare is a well‐documented barrier to transgender women's uptake of PrEP and primary healthcare (PHC). We aimed to demonstrate the utility of a PrEP quality improvement intervention in pinpointing and addressing barriers to PrEP use among transgender women in Vietnam.

**Methods:**

We applied a real‐world participatory continuous quality improvement (CQI) and Plan‐Do‐Study‐Act (PDSA) methodology to ascertain barriers to PrEP uptake among transgender women and determine priority actions for quality improvement. A CQI team representing transgender women leaders, key population (KP)‐clinic staff, public‐sector HIV managers and project staff applied PDSA to test solutions to identified barriers that addressed the primary quality improvement outcome of the monthly change in PrEP uptake among transgender women and secondary outcomes, including month‐3 PrEP continuation, the impact of offering PHC on PrEP uptake and unmet PrEP need. We utilized routine programmatic data and a descriptive cross‐sectional study enrolling 124 transgender women to measure these outcomes from October 2018 to September 2021.

**Results:**

Five key barriers to PrEP uptake among transgender women were identified and corresponding solutions were put in place: (1) offering gender‐affirming care training to KP‐clinics and community‐based organizations; (2) integrating gender‐affirming services into 10 KP‐clinics; (3) offering PHC through five one‐stop shop (OSS) clinics; (4) implementing a campaign addressing concerns related to hormone use and PrEP interactions; and (5) developing national HIV and transgender healthcare guidelines. New PrEP enrolment and month‐3 PrEP continuation increased significantly among transgender women. Of 235 transgender women who initially sought healthcare other than PrEP at OSS clinics, 26.4% subsequently enrolled in PrEP. About one‐third of transgender women reported unmet PrEP need, while two‐thirds indicated an interest in long‐acting cabotegravir.

**Conclusions:**

Offering gender‐competent, integrated PHC can increase PrEP enrolment and continuation, and can be an entry‐point for PrEP among those seeking care within PHC clinics. More work is needed to expand access to transgender women‐led and ‐competent healthcare in Vietnam.

## 1 INTRODUCTION

While no HIV prevalence or incidence estimates exist for transgender women who have sex with men in Vietnam, small urban samples have measured HIV prevalence from 16.0% to 18.0% [1, 2]. These rates are higher than sentinel HIV prevalence rates measured among men who have sex with men (13.4%), people who inject drugs (12.7%) and female sex workers (3.1%) in Vietnam within the same time period [3]. As in other countries, studies in Vietnam have found that transgender women who have sex with men may experience intersectional and reinforcing factors that increase the risk of HIV acquisition, including frequency of condomless receptive anal sex, sex work, poverty, violence, stigma, discrimination and social exclusion [4, 5, 6].

The Vietnam Ministry of Health (MOH) has developed and led a robust national HIV programme focused on reaching people who inject drugs, female sex workers and, since 2015, men who have sex with men [2]. Since 2020, the programme has recognized transgender women as distinct from men who have sex with men and has made HIV services designed to meet the specific needs of transgender women available in some localities [7].

Oral pre‐exposure prophylaxis (PrEP) services have been available in Vietnam since 2017 and can be accessed in 29 of Vietnam's 63 provinces, with 32,000 people using PrEP in 2021 [8]. PrEP services are delivered through a combination of public‐ and private‐sector clinics, including those owned and operated by key population (KP)‐led social enterprises. Efforts have been made to differentiate, demedicalize and simplify oral PrEP in Vietnam by making it available through mobile services (“PrEP Bus”), via telehealth and home‐delivery, and integrated into primary healthcare (PHC).

When PrEP was first initiated in Vietnam, gender‐affirming healthcare for transgender women was largely unavailable. During the first PrEP pilot in Vietnam, “Prepped for PrEP,” we found that while PrEP uptake increased five‐fold among men who have sex with men from the start to the end of the pilot (reaching 1069 PrEP users), there was no increase in monthly enrolment among transgender women, with only 62 enrolling over the 18 months of implementation [9]. The main reasons transgender women cited for not accessing PrEP were fear that PrEP antiretrovirals would interfere with or reduce the impact of feminizing hormones or would otherwise result in unwelcome side effects [9].

To better respond to transgender women's PrEP and healthcare needs, the US Agency for International Development (USAID)/PATH Healthy Markets project implemented a rapid needs assessment recruiting 409 transgender women in December 2018 from Hanoi and Ho Chi Minh City (HCMC) [10]. The median age of survey respondents was 23. The assessment found that while most (68.6%) transgender women participants knew about PrEP, only 7.6% had ever used or reported currently using PrEP. However, more than half (59.8%) of those not on PrEP stated that they wanted to use it, indicating a significant unmet need. The assessment also found that most (60.5%) transgender women wanted gender‐affirming care to be a part of their routine healthcare services. Overall, fear of mistreatment by staff and/or other patients for being transgender and feeling that healthcare providers are not comfortable or knowledgeable in providing care for transgender individuals were cited as major reasons for not seeking healthcare [10].

These findings led to an initiative by Healthy Markets to improve the quality of PrEP services reaching transgender women as part of integrative PHC in Vietnam through a continuous quality improvement (CQI) intervention utilizing the Plan‐Do‐Study‐Act (PDSA) methodology. This paper describes the actions taken and the key results and implications of that work. It aims to demonstrate the utility of a PrEP quality improvement intervention in pinpointing and addressing key barriers to PrEP use among transgender women in Vietnam.

## 2 METHODS

### 2.1 Application of a participatory quality improvement intervention

In response to the low monthly uptake of PrEP among transgender women noted by 2017–2018 Prepped for PrEP pilot and findings from the Healthy Markets project's 2018 transgender healthcare rapid needs assessment, this paper's authors applied a CQI approach to better understand and address barriers to care among transgender women. CQI best practice involves applying a participatory team approach to assessing and responding to quality challenges and utilizing complimentary data sources to inform the state of healthcare quality being measured, including perspectives from the people who use the services, routine programme data, service provision observation, medical record audits and in some cases, survey data [11]. PDSA can be used in CQI to systematically and iteratively assess the quality of care (often against standards), identify problem areas, generate possible solutions, test those solutions and adjust them over time through ongoing PDSA cycles [12]. PDSA, therefore, provides a framework for continued learning and measurement of change in healthcare quality through practical, real‐world experiments.

In 2017, we incrementally developed and adapted a tailored CQI approach for PrEP services. This work evolved into forming CQI teams around each clinic (*n* = 29) that included clinic health workers and managers, community PrEP users, local government HIV focal points and Healthy Markets staff. Of the 29 clinics, 10 were private clinics led and owned by KPs and primarily served men who have sex with men and transgender women. Their CQI efforts are described in this paper. Of these 10 clinics, two are located in Hanoi, the capital city of Vietnam; six in HCMC, the largest city in the country; and two in Dong Nai, a peri‐urban province adjacent to HCMC. These CQI efforts were ultimately encoded in a toolkit that has helped to systematize CQI‐PDSA approaches for all key HIV and PHC services [13].

To address the key challenge of low PrEP uptake among transgender women, we formed a specific group comprised of transgender women community leaders and organizations, 10 private KP‐led clinics and Healthy Markets staff (known from here on as the transgender women CQI healthcare team) to pinpoint key quality‐of‐care challenges (using a baseline quality assessment that reviews indicators from six data sources), determine how to address these challenges, put in place strategies and then track quantitative and qualitative data at designated time points to determine if the action or course correction was effective. From mid‐ to late‐2018, this involved organizing feedback sessions with transgender women PrEP users, assessing KP‐clinic standard operating procedures and staff capacity to offer gender‐affirming care, reviewing clinic data and assessing national guidance.

#### 2.1.1 Outcomes of interest

Our *primary* outcome of interest was the monthly change in PrEP uptake among transgender women over time (calculated as a monthly variation in new PrEP enrolment among transgender women). We defined our *secondary* CQI‐PDSA outcomes of interest as: (1) month‐3 PrEP continuation over time among transgender women (measured as a successful 3‐month refill after the first 3 months on PrEP); (2) whether offering integrated services might lead to secondary PrEP enrolment for transgender women accessing non‐PrEP services when first seeking care (measured through five KP‐led one‐stop shop [OSS] clinics’ service use data); (3) degree of unmet PrEP need among transgender women and the role a new PrEP product, namely long‐acting cabotegravir (CAB‐LA), might play in influencing PrEP use decision‐making.

Related to measuring unmet PrEP need, we utilized data from a February 2021 cross‐sectional survey on KP preferences, use of, and willingness to pay for HIV and PHC services. The study enrolled 124 transgender women in Hanoi, HCMC, Can Tho and Dong Nai Provinces. An ethics review was provided by the Institute of Social and Medical Studies Institutional Review Board in Hanoi. Oral informed consent was obtained from all participants. Preliminary PrEP‐focused results from this study have been published in brief elsewhere [14].

### 2.2 Data capture and analysis

To assess changes in PrEP uptake among transgender women following implementation of the above change strategies, we utilized programmatic HIV service data from 10 KP‐led PrEP clinics (including five designated as integrated PHC OSS clinics—see below) supported through Healthy Markets. These data are reported by clinics monthly through a secure online system. Specific programmatic data from the five OSS clinics were assessed to explore transgender women's use of PrEP and other services from October 2020 to September 2021. We were interested in whether offering integrated person‐centred care services might lead to secondary PrEP enrolment for transgender women.

Programmatic PrEP and OSS services data (including HIV testing; sexually transmitted infection, hepatitis B and C, and hormone testing/services; and mental healthcare) were cleaned and analysed monthly based on Healthy Markets project monitoring protocols. Data quality was verified based on routine project data quality assurance procedures. The data were used to calculate transgender women's cumulative and monthly changes in PrEP enrolment over time, month‐3 PrEP continuation, OSS service utilization and secondary PrEP enrolment.

For the cross‐sectional survey, data were collected through individual interviews and tablet recordings (CAPI) using CSPro software. Data were transferred to Stata v14.0 for cleaning and analysis. The data collectors synthesized the data daily, after which the data management team conducted a data quality assessment. If data were unavailable, missing or unusual (based on the general local trends), the data management team contacted the clinics providing the data the same day to determine the reasons behind data gaps or inconsistencies and then correct them. Descriptive statistics were used to calculate PrEP use and unmet need. See Table 1 for a description of these methods.

**Table 1:** Data methods

No.	Method	Description	Linked outcomes and results
1	Continuous quality improvement (CQI)—Plan‐Do‐Study‐Act (PDSA) cycles	CQI teams applied PDSA to test solutions to identified barriers in pre‐exposure prophylaxis (PrEP) uptake among transgender women. This process includes an iterative cycle of assessment, action planning and testing actions to address quality challenges: (1) gathering data from six information sources at each clinic (i.e. a clinic self‐assessment, observation of service delivery and clinic practices, file audit, routine performance data, infrastructure check and direct client feedback); (2) developing action plans for addressing these challenges; and (3) tracking quantitative and qualitative data at designated time points to determine if the action or course correction was effective.	Key barriers to PrEP uptake and quality improvement actions needed
2	Routine programmatic data analysis	HIV service data from 10 KP‐led PrEP clinics were reported by clinics monthly through a secure online system and assessed by the study team to explore transgender women's use of PrEP and other services from October 2020 to September 2021.	Cumulative and monthly changes in PrEP enrolment over time, month‐3 PrEP continuation, one‐stop shop service utilization and secondary PrEP enrolment
3	Descriptive cross‐sectional study	Data were collected through individual interviews and tablet recordings using CSPro software and descriptive statistics were used to calculate the main and secondary outcomes of interest for CQI‐PDSA efforts.	PrEP use and unmet PrEP need; preferences for use of long‐acting injectable cabotegravir

Abbreviations: CQI, continuous quality improvement; KP, key population; PDSA, Plan‐Do‐Study‐Act; PrEP, pre‐exposure prophylaxis.

## 3 RESULTS

### 3.1 Key barriers identified through CQI

Through the baseline quality assessment, the transgender women CQI healthcare team identified five fundamental barriers to PrEP uptake among transgender women to address through PDSA: (1) limited knowledge of transgender‐competent healthcare among healthcare providers; (2) limited roles of transgender women in health service provision within private‐ and KP‐clinics; (3) lack of gender‐affirming care integrated into PHC services in PrEP clinics and related, absence of guidelines to inform *how* to integrate gender‐affirming care; (4) limited information on transgender women community norms around drug–drug interaction between PrEP and gender‐affirming hormones; and (5) lack of national guidelines on gender‐affirming care as part of HIV services (Table 2).

**Table 2:** Key barriers to care and Plan‐Do‐Study‐Act steps taken to enable greater access and uptake

No.	Major barriers to PrEP/PHC	Quality improvement actions to enable greater PrEP/PHC access
1	Limited knowledge of transgender‐sensitive and transgender‐competent healthcare among healthcare providers.	**Training**. Improved PrEP clinic healthcare provider competencies in gender‐affirming care through training and mentoring by Tangerine Academy and reinforced through local learning.
2	Limited roles of transgender women in care delivery within private and KP clinics.	**Staffing**. Transgender women lay healthcare workers were employed by clinics and trained to provide dedicated counselling and support to clients.
3	Lack of gender‐affirming or adequately integrated primary healthcare services in clinics offering PrEP.	**Service**. Offered gender‐affirming hormone‐level testing, counselling and referrals for feminizing procedures, and additional integrative primary healthcare services through private clinics.
4	Limited information on transgender women's community norming around drug–drug interaction between PrEP and gender‐affirming hormones.	**Outreach**. Implemented a transgender women‐led communications campaign on PrEP use and hormones to address concerns expressed by the community.
5	No national guidelines on gender‐affirming care as part of HIV services.	**Guidelines**. Assisted the Vietnam Ministry of Health in developing first‐ever transgender HIV and healthcare guidelines as a result of actions 1–4.

Abbreviations: KP, key population; PHC, primary healthcare; PrEP, pre‐exposure prophylaxis.

### 3.2 Quality improvement actions

Below (and in Table 2), we describe the quality improvement actions taken to address these barriers.

#### 3.2.1 Training

In August 2018, the Tangerine Academy, a training branch of the Institute of HIV Research and Innovation in Bangkok, Thailand, provided in‐person trainings on transgender‐competent care to 20 healthcare providers from KP‐clinics in Hanoi and HCMC, representatives from transgender women‐led community‐based organizations (Ruby, Strong Lady and Venus), and members of the HCMC and Hanoi Provincial AIDS Committees. The training oriented healthcare providers and local health leaders on gender‐sensitive and ‐affirming care, management of gender‐affirming hormone treatment and service provision, and strategies to integrate gender‐affirming care into HIV services.

The Tangerine Academy then provided on‐site technical assistance, followed by remote mentoring for clinic staff on interpretation of laboratory results and management of hormone treatment with PrEP. A second on‐site visit was conducted in late 2019 to mentor clinics in Hanoi. The Tangerine Academy also participated in a meeting with the MOH to inform the development of national HIV guidelines focused on the needs of transgender individuals. Subsequent online training was held in August 2021 for more than 90 people, including 40 healthcare providers from 14 clinics, 30 staff from transgender women‐led community‐based organizations and participants from the MOH.

#### 3.2.2 Staffing

Although clinics led and staffed by KPs in HCMC and Hanoi existed in 2018, they had no adequately defined and dedicated roles specifically for transgender women providers. The transgender women CQI healthcare team supported the placement of transgender women healthcare providers in the participating KP‐led PHC clinics.

#### 3.2.3 Service

In October 2018, five KP‐led PHC PrEP clinics (Alocare, Bien Viet, Galant, Glink and My Home) began offering estradiol and testosterone hormone‐level testing, and counselling and referrals for feminizing procedures (of which there are limited options in Vietnam). The KP‐led clinics then further expanded their service offerings by adding sexually transmitted infection testing and treatment, viral hepatitis services, and mental health assessments and counselling, alongside the existing HIV services (i.e. testing, antiretroviral therapy, care for coinfections, and PrEP and non‐occupational post‐exposure prophylaxis). These clinics were designated as OSS clinics in 2020 and received additional financing through USAID/US President's Emergency Plan for AIDS Relief to offer an expanded service package to clients. Five additional KP‐clinics were opened from late 2018 to 2021 and provided with training and standard operating procedures on gender‐affirming PrEP service delivery.

#### 3.2.4 Outreach

The Healthy Markets project assisted the Vietnam Network of Transgender People, transgender women influencers and KP‐led private clinics to launch a dedicated campaign known as “Be Me, Be Happy” that focused on creating an inclusive online and offline community for information and support on healthcare and wellbeing among transgender women. Through this campaign, transgender women leaders (including the co‐authors of this paper) aimed to directly address hormone–PrEP drug interaction concerns through specific online content on the “Be Me, Be Sexy” Facebook page (with 21,000 followers), video clips, a TikTok PrEP ambassador championship and small‐scale engagements that enable transgender women considering PrEP to ask questions to peer transgender women PrEP experts and healthcare workers.

#### 3.2.5 Guidelines

As a result of the described quality improvement efforts undertaken, the Vietnam Administration for HIV/AIDS Control (VAAC) expressed interest in developing national guidelines on transgender healthcare as part of HIV services. Healthy Markets, with technical engagement from Tangerine Academy, and transgender leaders (including the co‐authors of this paper) partnered with the VAAC to develop and publish these guidelines in 2020.

### 3.3 Key outcomes

#### 3.3.1 PrEP uptake

**Figure 1:**
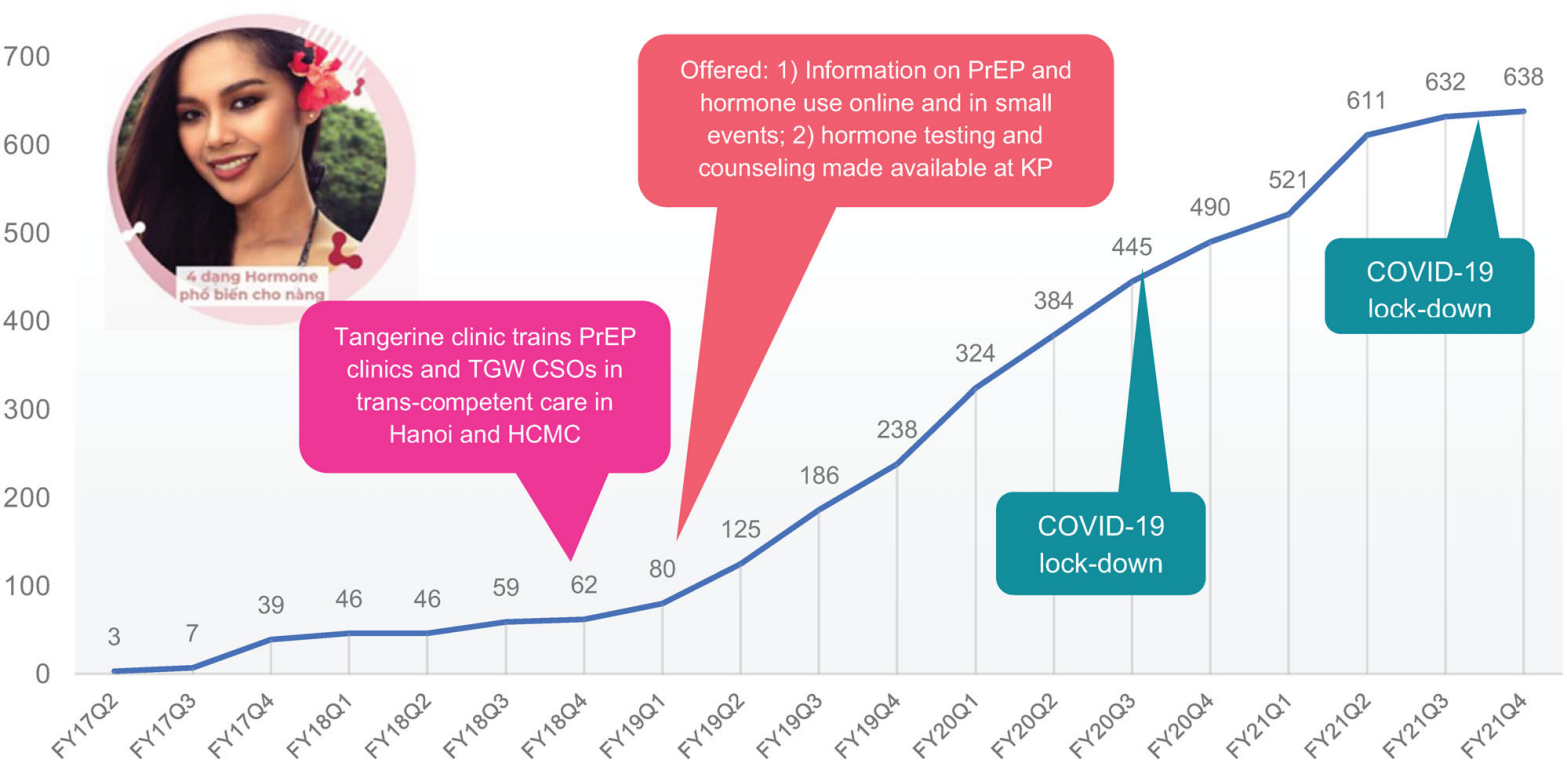
Transgender women's pre‐exposure prophylaxis uptake before and after the introduction of gender‐affirming services and impact of COVID‐19 on enrolment. Abbreviations: CSO, civil society organization; HCMC, Ho Chi Minh City; KP, key population; PrEP, pre‐exposure prophylaxis; TGW, transgender women.

The cumulative number of transgender women ever enrolled in PrEP increased from 46 in 2018 to 638 in 2021, with 485 using PrEP from October 2020 to September 2021. Cumulative transgender women's PrEP uptake increased from 3.4 new enrolments per month on average from 2017 through 2018 to 20 per month on average from 2019 through 2020, during the implementation of the five described CQI interventions (Figure 1). New enrolment rates declined substantially from April through September 2021 as strict COVID‐19 lockdown orders were enforced [15, 16].

#### 3.3.2 Month‐3 PrEP continuation

**Figure 2:**
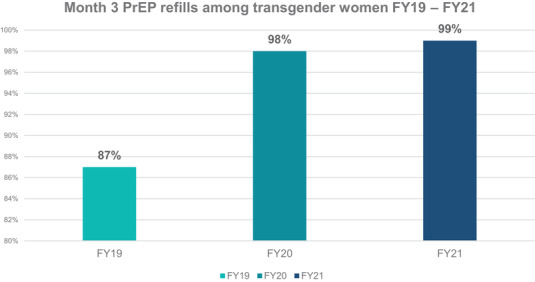
Month‐3 pre‐exposure prophylaxis continuation among transgender women in the fiscal year 2019 (October 2019–September 2020), the fiscal year 2020 (October 2020–September 2021) and the fiscal year 2021 (October 2020–September 2021). Abbreviation: FY, fiscal year.

Overall, month‐3 PrEP continuation among transgender women increased from 87% during October 2018 through September 2019 to 98% during October 2019 through September 2020 to 99% during October 2020 through September 2021 (Figure 2). These represented the highest continuation rates among all other KP groups using PrEP.

#### 3.3.3 Impact of integrated PHC services on PrEP uptake

**Figure 3:**
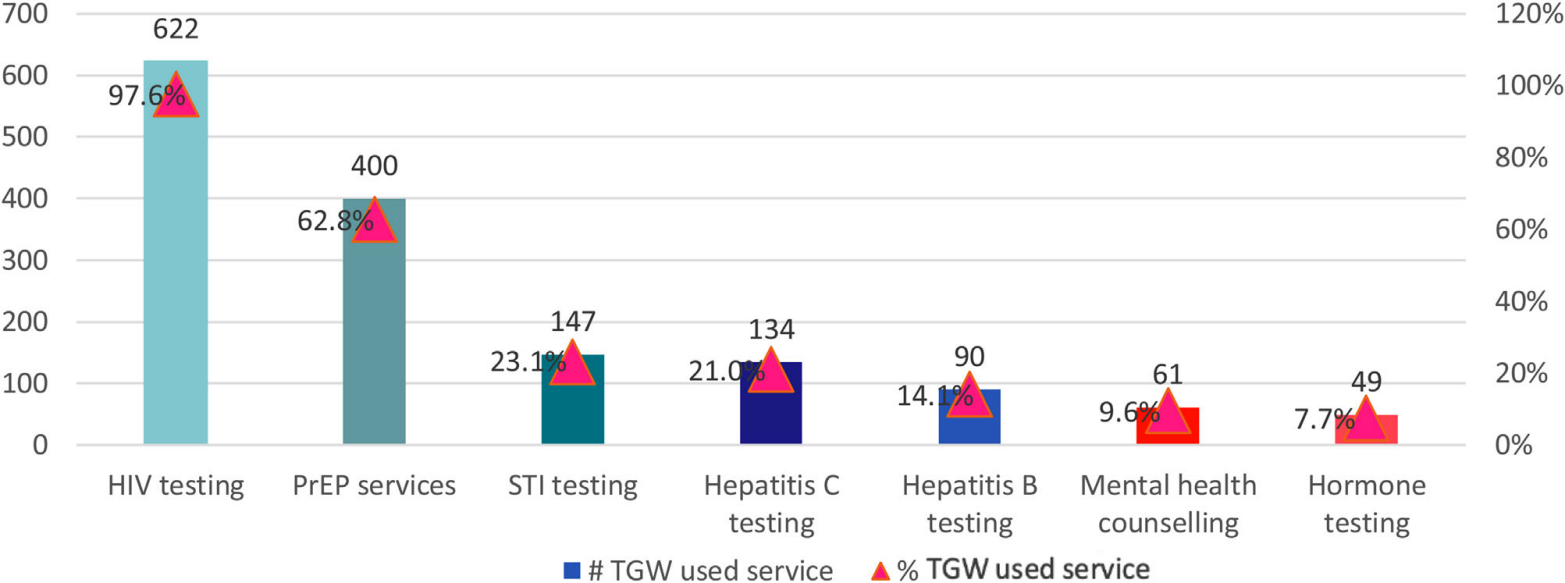
Services used by transgender women at five key population‐led one‐stop shop primary healthcare clinics, October 2020–September 2021 (number of clinic visits). Abbreviations: PrEP, pre‐exposure prophylaxis; STI, sexually transmitted infection; TGW, transgender women.

From October 2020 through September 2021, 416 transgender women sought healthcare at one of the five OSS clinics, representing 637 total visits. The median age among transgender women seeking OSS healthcare services was 29 (range 15–49). The majority of transgender women (96.6%, *n* = 402) sought services in HCMC, while 14 did so in Hanoi. The most frequent services transgender women used at the OSS clinics were HIV testing (97.6%) and PrEP services (62.8%), followed by sexually transmitted infection testing (23.1%), hepatitis C testing (21.0%) and hepatitis B testing (14.1%). Mental health counselling and hormone testing were accessed less frequently (Figure 3).

Among the 235 transgender women who sought healthcare other than PrEP at the OSS clinics, 62 (26.4%) subsequently enrolled in PrEP services. Transgender women who were of older age (OR = 5.21, 95% CI 2.05–13.39, *p* <0.001) and who sought PrEP offline (through word‐of‐mouth, direct peer referrals or attending an event) (OR = 2.29; 95% CI 1.20–4.44, *p* = 0.007) had greater odds of PrEP services being the initial/primary reason they attended an OSS clinic (Table 3). Among this population, 41% subsequently used other OSS health services.

**Table 3:** Unadjusted factors associated with secondary pre‐exposure prophylaxis enrolment at one‐stop shop clinics

		Transgender women who sought PrEP first	Transgender women who sought PrEP only after receiving other services	Univariate logistic regression
Age group	25–49	168 (79.3%)	44 (20.7%)	OR = 0.19, 95% CI 0.07–0.49, *p* <0.001
	15–24	11 (42.3%)	15 (57.7%)	
Sources	Offline only	100 (82.6%)	21 (17.4%)	OR = 2.29, 95% CI 1.20–4.44, *p* = 0.007
	Both offline and online	79 (67.5%)	38 (32.5%)	

Abbreviations: CI, confidence interval; OR, odds ratio. PrEP, pre‐exposure prophylaxis.

#### 3.3.4 Unmet PrEP need

Thirty‐one percent (*n* = 39) of transgender women reported being on PrEP, 28.2% (*n* = 35) wanted to take PrEP but not having done so yet, 26.2% had heard of CAB‐LA and 63.4% indicated an interest in using CAB‐LA over oral PrEP. The primary reason stated for wanting CAB‐LA over oral PrEP was not needing to remember to take pills (66.9%); conversely, the primary reason for opting for oral PrEP over CAB‐LA was not liking injections (61.8%).

## 4 DISCUSSION

We found that by applying collaborative CQI‐PDSA approaches, we were able to pinpoint five primary barriers to transgender women's PrEP uptake and put in place actions to address them. After applying these programmatic change actions, we saw an overall 17‐fold monthly increase in PrEP uptake among transgender women from the initial Prepped for PrEP pilot and measured a 12% increase in month‐3 PrEP continuation among transgender women from the end of 2018 to September 2021.

The participants described in this paper represent the majority of transgender women on PrEP in Vietnam. The national PrEP programme reported a total of 32,000 people on PrEP from October 2020 to September 2021; of these, 574 (2%) identify as transgender women. The 10 KP‐clinics described in this paper enrolled 485 transgender women on PrEP during the same time period, representing 84.5% of the national total [15].

Offering integrated gender‐affirming PHC may have supported the increase in PrEP continuation. van Griensven et al. reported that offering transgender‐specific integrated PHC services resulted in increased PrEP enrolment and retention [16]. Additionally, we found that offering integrated services led to secondary PrEP enrolment of just over one‐quarter of the transgender women seeking other healthcare services at OSS clinics. We also found that about one‐quarter of transgender women first seeking PrEP at OSS clinics subsequently utilized other PHC services at the clinics. However, we noted lower than anticipated utilization in mental health, sexually transmitted infection and hormone testing services at these clinics. These services were intended to be offered on a greater scale starting from late 2019 but with COVID‐19 surges, training, clinical mentoring and in‐clinic care seeking were curtailed. With the adult COVID‐19 vaccination rate near 96% and the country has opened up from May 2022, training and support to OSS clinics to intensify and strengthen these services has begun. Importantly, the OSS model is delivered by KP‐owned and ‐led private clinics that have diversified their income streams and are able to sustain these services.

While CQI‐PDSA efforts described in this paper equipped KP‐led clinics to offer transgender‐sensitive and ‐competent care, a critical next step will be the establishment of transgender‐led and ‐owned clinics providing differentiated care [17, 18]. The 2020 MOH transgender HIV services guidelines will be vital in enabling comprehensive gender‐affirming services to be available on a larger scale.

Though the described improvements in the quality of gender‐affirming care have been important, there is still an unmet PrEP need among transgender women in the country. Nearly, a third of individuals in the cross‐sectional study reported wanting PrEP but not yet seeking it. While this is lower than the reported unmet PrEP need (56%) reported in the December 2018 needs assessment, it is still significant [10]. It will be very important to understand the underlying root causes of this unmet need. Moreover, given about two‐thirds of transgender women indicated an interest in taking CAB‐LA over oral PrEP, it will be essential to understand potential challenges and opportunities in the use of CAB‐LA among transgender women and ensure its integration into gender‐affirming services.

COVID‐19 has had a profound impact on the overall wellbeing of transgender women in Vietnam [19, 20]. During the worst part of the lockdown (April–September 2021), 46.2% of transgender women stopped using PrEP at Healthy Markets‐supported clinic sites [21]. Efforts are underway by community organizations and clinics to offer economic relief and re‐engage transgender women in PHC services, including PrEP.

There are several limitations associated with the data presented in this paper. First, our CQI‐PDSA methodology was not able to directly isolate the linear impact of the CQI‐PDSA interventions on the increased PrEP uptake we observed. Second, the sample size and very limited number of programmatic socio‐demographic factors routinely collected (age and online or offline PrEP enrolment) limited robust multivariable analysis for exploring factors associated with PrEP uptake. Third, our work was not based on a probability sample nor necessarily representative of transgender women in HCMC, Hanoi and Dong Nai so cannot be interpreted as such. Last, with limited resources, we were not able to address the needs of transgender men and gender‐nonbinary individuals who might be interested in PrEP. This will be an important area of focus in Vietnam's next phase of PrEP scale‐up.

Despite these limitations, the increase in transgender women enrolling in PrEP after the five primary quality improvement actions were put in place is encouraging. The improvements in month‐3 PrEP continuation are equally reassuring. Further, our paper included data from 85% of transgender women using PrEP in Vietnam, allowing for confidence in interpreting results.

## 5 CONCLUSIONS

Using a participatory CQI‐PDSA approach was critical to identifying underlying barriers to transgender women's uptake of PrEP services and significantly increasing new PrEP uptake, month‐3 continuation and secondary PrEP enrolment through other PHC services. Our findings suggest that more investment and resources are needed to increase transgender‐sensitive and ‐competent care in Vietnam, including through transgender‐led and ‐owned clinics, and to support the implementation at scale of the 2020 MOH transgender HIV services guidelines. Another vital next step will be to expand PHC services to include a wider array of gender‐affirming care and address the needs of transgender men and gender‐nonbinary individuals. Intensive work will also be needed to enable transgender communities to recover from COVID‐19 impacts. By taking these actions, the Vietnam MOH will be better placed to meet its goals for HIV epidemic control and universal health coverage by 2030.

## COMPETING INTERESTS

The authors have no competing interests to declare.

## AUTHORS’ CONTRIBUTIONS

AHD and KEG conceived the paper and wrote the manuscript in equal parts. CMHV and TTN provided leadership in all CQI‐PDSA efforts. HTTP provided national leadership for CQI‐PDSA and the development of the national guidelines. RJ led training and mentoring described in the manuscript and contributed to the writing of the paper. AHD, KEG, BNV and TTT guided CQI‐PDSA efforts on behalf of USAID/PATH Healthy Markets. CMHV, TTN, TML and KQD oversaw the delivery of transgender‐affirming care in KP‐clinics. TMN and LB provided technical input and strong commitment to the improvement of PrEP services for transgender women. LKT led programmatic and descriptive study data analytics and contributed to the writing of the paper. ZH provided copy editing and coordination.

## FUNDING

Financing for the work described in this article was made possible by the US Agency for International Development, through the US President's Emergency Plan for AIDS Relief.

## Data Availability

The data that support the findings of this study are available from the corresponding author upon reasonable request.
